# Supplementary material for: Sepsis and delayed cerebral ischemia are associated and have a cumulative effect on poor functional outcome in aneurysmal subarachnoid hemorrhage
Source: Front Neurol. 2024 May 31;15:1393989. doi: 10.3389/fneur.2024.1393989 (PMC11179438; doi:10.3389/fneur.2024.1393989)
Supplement: Supplementary file 1 [file Table_1.DOCX]

Supplementary Material

# Supplementary Text

**Text S1.** **Clinical management of aSAH patients.**

Clinical management of patients with aSAH was performed as described previously (21) and the procedures followed were in accordance with institutional guidelines. A cerebral CT scan including CT-angiography (CTA) was initiated immediately after admission. Patients who were unable to protect their airway due to a decreased level of consciousness were intubated. Unconscious patients with space-consuming subdural hematoma or intracerebral hemorrhage and/or acute hydrocephalus or intraventricular hemorrhage were immediately treated surgically, i.e., hematoma removal, aneurysm repair, and, in selected cases, decompressive craniectomy. External ventricular drains (EVD) were placed via the Kocher point and in a tunneled fashion. Before aneurysm repair, systolic blood pressure was kept <140 mmHg. Decision on the modality of aneurysm repair was made in interdisciplinary consensus. Within the first 6–24 hours after aneurysm repair, systolic blood pressures were maintained between 120 and 180 mmHg. After 24 hours or following the exclusion of re-hemorrhage after aneurysm repair, spontaneous systolic blood pressures were permitted, but generally maintained above 130 mmHg. Patients routinely received nimodipine 6 × 60 mg orally for at least 15 days following aneurysm repair.

From January 2016 an updated protocol for standardized detection and management of DCI was implemented by the department of neurosurgery in line with our previous studies (22, 23), including thorough neurological examinations every 2–4 hours in combination with a CT-perfusion (CTP) screening protocol, especially for intubated and sedated patients, who were thereby not neurologically assessable. CTP measurements were performed on admission, 6–12 hours after aneurysm repair, on day 3 or 4, as well as on day 9 to 11 after SAH ictus. Additionally, digital subtraction angiography (DSA) was performed on admission and on day 6 to 9 after the ictus. Patients who developed clinical features of DCI underwent CT, CTA, and CTP. If mean transit time in CTP was >1.5 times baseline, DSA was indicated. In case of persistent clinical or radiological features of DCI, patients were treated according to a standardized, escalating treatment protocol: 1. induced hypertension with a targeted systolic blood pressure of >180 mmHg; 2. solitary intra-arterial nimodipine bolus applications during DSA; and 3. angiographic application of an intra-arterial catheter for continuous nimodipine administration over 48 hours with CTP imaging in between each escalating step.

Before January 2016, management decisions were usually taken on an individual basis and patients were predominately treated according to the presence of angiographic vasospasm. Patients were neurologically assessed in the ICU and underwent DSA on days 6 to 9 after aneurysm repair or immediately upon clinical deterioration for assessment of the presence of angiographic vasospasm. Induced hypertension was not applied as consistently. Patients with severe vasospasm and/or clinically relevant vasospasm or patients with progressive vasospasm on repeated DSA received an intra-arterial catheter for continuous nimodipine administration as first-line treatment. Angiographic vasospasm was monitored by a follow-up DSA after 72 h. We adjusted for these changes in the DCI treatment protocol during all our analyses by incorporating a binary (yes/no) factor variable (DCI protocol).

# Supplementary Tables

| Table S1. Reasons for exclusion during neuroradiological validation. | |
| --- | --- |
| Reason | **N** |
| Spontaneous SAH, no aneurysm | 14 |
| Traumatic SAH | 14 |
| Initial SAH not in study period, ICD-coding due to follow-up | 9 |
| No CT scan of initial SAH available | 6 |
| SAH initially treated elsewhere | 6 |
| Ischemic stroke with SAH component | 5 |
| Intracerebral bleeding with SAH component | 5 |
| No SAH | 3 |
| SAH already consolidated in initial CT | 2 |
| SAH associated with cerebral neoplasia | 2 |
| Arterio-venous malformation with SAH component | 2 |
| SAH as surgical complication | 2 |
| Subdural hematoma with SAH component | 1 |
| SAH associated with severe coagulation abnormalities due to |  |
| ECMO therapy | 5 |
| cardiac resuscitation | 3 |
| sepsis | 3 |
| cerebritis | 1 |
| Missing data | 1 |
|  |  |
| Total | 84 |
| CT, computed tomography; ECMO, extracorporeal membrane oxygenation; ICD, international classification of diseases; SAH, subarachnoid hemorrhage. | |

| Table S2. Sensitivity analysis: multivariable logistic regression model (N_sens_=221) for delayed cerebral ischemia (DCI) excluding patients who developed sepsis before DCI (N_excluded_=17). | |
| --- | --- |
| Variable | **aOR [95%CI]; p-value** |
| Age | 1.00 [0.97-1.02]; 0.85 |
| Smoking | 0.62 [0.32-1.19]; 0.15 |
| WFNS I-III vs. IV+V | 0.89 [0.46-1.69]; 0.71 |
| Clipping | 1.08 [0.59-2.00]; 0.80 |
| Sepsis | 0.85 [0.37-1.95]; 0.70 |
| To ensure statistical robustness, because only nine patients developed sepsis after DCI, the number of variables and their respective levels in the model were reduced. Thus, WFNS was dichotomized according to Abdulazim et al. (21). aOR, adjusted odds ratio; DCI, delayed cerebral ischemia; EVD, external ventricular drainage; WFNS, World Federation of Neurological Surgeons SAH grading scale. | |

| Table S3. Multivariable logistic regression model for functional outcome (mRS 0-3 vs. 4-6). | |
| --- | --- |
| Variable | **aOR [95% CI]; p-value** |
| Age | **1.84 [1.29-2.62];** **<0.01** |
| Smoking | 1.08 [0.53-2.18]; 0.83 |
| WFNS grade II | 0.48 [0.19-1.23]; 0.13 |
| III | 0.39 [0.08-1.90]; 0.24 |
| IV | 0.53 [0.19-1.45]; 0.21 |
| V | 1.94 [0.66-5.67]; 0.23 |
| Clipping | 1.89 [0.95-3.77]; 0.07 |
| EVD placement | **21.87 [6.76-70.77]; <0.01** |
| DCI protocol | 0.68 [0.29-1.56]; 0.36 |
| DCI | **2.45 [1.18-5.07]; 0.02** |
| Sepsis | **2.85 [1.23-6.63]; 0.02** |
| Bold text indicates statistical significance at p<0.05 level. aOR, adjusted odds ratio; DCI, delayed cerebral ischemia; EVD, external ventricular drainage; mRS, modified Rankin Scale; WFNS, World Federation of Neurological Surgeons SAH grading scale. | |

| Table S4. Sensitivity analysis including comparative statistics of multivariable logistic regression model (N=238) additionally adjusted for interaction between sepsis and DCI regarding functional outcome (mRS 0-3 vs. 4-6). | |
| --- | --- |
| Variable | **aOR [95% CI]; p-value** |
| Intercept | **0.06 [0.02-0.23]; <0.01** |
| Age | **1.86 [1.29-2.67]; <0.01** |
| Smoking | 1.22 [0.59-2.49]; 0.59 |
| WFNS grade II | 0.55 [0.21-1.44]; 0.23 |
| III | 0.42 [0.08-2.13]; 0.30 |
| IV | 0.57 [0.21-1.60]; 0.29 |
| V | 2.32 [0.78-6.91]; 0.13 |
| Clipping | 2.01 [1.00-4.03]; 0.05 |
| EVD placement | **25.74 [7.39-89.59]; <0.01** |
| DCI protocol | 0.60 [0.25-1.41]; 0.24 |
| DCI | 1.58 [0.71-3.53]; 0.27 |
| Sepsis | 1.38 [0.51-3.72]; 0.53 |
| Interaction term (DCI*sepsis) | **11.05 [1.31-93.27]; 0.03** |
|  |  |
| Model statistics |  |
| Akaike information criterion (AIC) | 246.06 |
| Bayesian information criterion (BIC) | 291.20 |
| Pseudo R-squared | 0.49 |
| Bold p-values indicate statistical significance at the p<0.05 level. aOR, adjusted odds ratio; DCI, delayed cerebral ischemia; EVD, external ventricular drainage; mRS, modified Rankin Scale; WFNS, World Federation of Neurological Surgeons SAH grading scale. | |
